# Characteristics of T-cell receptor repertoire of stem cell-like memory CD4+ T cells

**DOI:** 10.7717/peerj.11987

**Published:** 2021-08-25

**Authors:** Shiyu Wang, Longlong Wang, Yang Liu, Yonggang Zhu, Ya Liu

**Affiliations:** 1 College of Life Sciences, University of Chinese Academy of Sciences, Beijing, China; 2 BGI-Shenzhen, Shenzhen, China; 3 China National GeneBank, BGI-Shenzhen, Shenzhen, China; 4 Shenzhen Key Laboratory of Single-Cell Omics, BGI-Shenzhen, Shenzhen, China; 5 School of Mechanical Engineering and Automation, Harbin Institute of Technology, Shenzhen, Shenzhen, China

**Keywords:** T-cell receptor beta chain repertoire, Complementarity determining region 3, CD4+ memory T cell, Stem-cell like CD4+ memory T cell, Public clonotypes

## Abstract

Stem cell-like memory T cells (Tscm) combine phenotypes of naïve and memory. However, it remains unclear how T cell receptor (TCR) characteristics contribute to heterogeneity in Tscm and other memory T cells. We compared the TCR-beta (TRB) repertoire characteristics of CD4+ Tscm with those of naïve and other CD4+ memory (Tm) in 16 human subjects. Compared with Tm, Tscm had an increased diversity across all stretches of TRB repertoire structure, a skewed gene usage, and a shorter length distribution of CDR3 region. These distinctions between Tscm and Tm were enlarged in top1000 abundant clonotypes. Furthermore, top1000 clonotypes in Tscm were more public than those in Tm and grouped in more clusters, implying more epitope types recognized by top1000 clonotypes in Tscm. Importantly, self-reactive clonotypes were public and enriched in Tscm rather than Tm, of type one diabetes patients. Therefore, this study highlights the unique features of Tscm different from those of other memory subsets and provides clues to understand the physiological and pathological functions of Tscm.

## Introduction

Memory T cells play the central role in coordinating innate and adaptive immune responses (MacLeod et al., 2009). After encountering antigens, naïve T cells differentiate to memory subsets and terminally differentiated effector T cells. During the differentiation process, T cells acquire functions and lose self-renewal abilities (Seita & Weissman, 2010). Following the theory of a hierarchical system of memory, naïve transits to central memory (Tcm) and effector memory (Tem) T cell subsets in turn (Fearon et al., 2006). Tcm is relatively long-lived, and a notion considers the stem cell-like characteristics of Tcm. However, the finding of stem cell-like memory (Tscm) challenges this notion (Gattinoni et al., 2011; Lugli et al., 2013). Tscm, a rare memory subset defined from naïve, is a long-term memory subset with self-renew and the plasticity to differentiate into other memory subsets and effector (Ahmed et al., 2016; Stemberger et al., 2009).

Recently, observations in diseases and vaccines unveil physiological and pathogenies functions of Tscm. In mice models, deleting T cells and transplanting Tscm could re-build the memory T cell population, including central and effector memory (Simons & Clevers, 2011). In clinical studies, human Tscm cells from naïve precursors enrich early after hematopoietic stem cell transplantation (HSCT), and contribute to peripheral reconstitution by differentiating into effectors (Cieri et al., 2015; Roberto et al., 2015). Stimulations with CMV, influenza vaccine and WT1 tumor antigen activate cytokine expression in part of these naïve-derived Tscm, suggesting that naïve specific to given antigens differentiate to Tscm (Roberto et al., 2015). Furthermore, HIV (Ahmed et al., 2016), smallpox, and yellow fever vaccines studies (Gattinoni et al., 2017) presented that antigen-specific CD8^+^ Tscm persisted in donors receiving vaccines after a long time, indicating that Tscm acts as a reservoir for maintaining these exogenous factors antigen-specific TCRs. In addition, CD4^+^ Tscm may involve in graft-versus-host disease (GVHD) and autoimmune disease. As shown by Jimbo et al. (2019), the peripheral proportion of CD4+ Tscm increased in graft-versus-host disease (GVHD) patients compared with no GVHD patients after HSCT. An increased proportion of CD4^+^ or CD8^+^ Tscms has also been observed in patients with autoimmune disease (Jimbo et al., 2019), such as systemic lupus erythematosus (SLE) (Lee et al., 2018), type 1 diabetes (Vignali et al., 2018), aplastic anaemia (Hosokawa et al., 2016), immune thrombocytopenia (Cao et al., 2019) and rheumatoid arthritis (Cianciotti et al., 2020). Recently, T-cell immunotherapies based on Tscm have been developed against HIV and cancer (Flynn & Gorry, 2014). However, in addition to antigen-driven, cytokines also involve in T cell differentiation. Interleukin-7 and Interleukin-15 combined with stimulation *via* CD3 and CD28 facilitate the differentiation of naïve to Tscm *in vitro* (Cieri et al., 2013). PD-L1 and TGF-b promote the differentiation from naïve to regular T cell (Batra et al., 2020). IFN-β can regulate the expansion of CD4^+^ memory T and NK cells to facilitate the anti-tumor effects of a novel form of 4-1BBL (Barsoumian et al., 2019). Therefore, these studies in infectious and autoimmune diseases raise interest in the specificity of TCR clonotypes enriched in Tscm and whether the TCR repertoire of Tscm is different from other memory subsets.

Individual has enormous diversity of TCR repertoire including over 10^6^ clonotypes (Qi et al., 2014; Soto et al., 2020). Complementary-determining region 3 (CDR3) is the most diverse part of TCR, and contribute to antigen recognition ability of TCR. The diversity of TCR repertoire is trimmed by inherent and exogenous factors. For naive, genetics (Gao et al., 2019; Posnett, 1995), the rearrangements of V(D)J segments, and thymus selection (Khosravi-Maharlooei et al., 2019) shape its TCR repertoire. For memory, both genetics and environment factors trim its TCR repertoire composition (Hou et al., 2020; Krishna et al., 2020). Previous studies suggest that antigen-specific clonotypes unevenly distributed among memory subsets. Acute infection-related clonotypes enriched in Tcm, while chronic infection-related clonotypes and autoimmunity related clonotypes maintained in Tem (Devarajan & Chen, 2013). In COVID19 patients, CD4^+^ responding clonotypes were biased to be expanded in Tcm more than in Tem (Minervina et al., 2021). Furthermore, a study using the transfer of genetically-modified virus-specific T cells showed that antigen-specific clonotypes only maintained in Tscm rather than other subsets after a long time (Roberto et al., 2015), suggesting that composition of TCR repertoire may different among memory subsets. in addition, T-cell differentiation However, it is still difficult to conclude that memory subsets have different composition of TCR clonotypes, because of the limitations of methods for screening antigen-specific TCRs.

High-throughput sequencing of TCR repertoire (TCR-seq) has become an essential technique in immunology. Recently, this technique is used to unveil the process of TCRs’ development in the thymus (Khosravi-Maharlooei et al., 2019), to promote the understanding of positive and negative selections, and to define the disease biomarkers (Liu et al., 2019). By TCR-seq, the differences of the TCR repertoire were shown among T cell subsets. The TCR beta chain (TRB) repertoire of CD4^+^ memory T cells has a shorter distribution of CDR3 length and a skewed V-gene usage, compared with that of Tn in peripheral blood. A study with three subjects shows that Tcm has a power law exponent higher than Tem (Oakes et al., 2017). It suggests a lower clonal expansion in Tcm. A model by the power law distribution was employed to separate type one diabetes from heathy donors based on the TRB repertoire of Tscm, but not that of Tcm (Koch et al., 2018). Furthermore, T cell receptor antigen specificity prediction methods based on the TRB CDR3 sequence have been developed recently (Zhang et al., 2020), and clonotypes targeting the same antigens can be clustered by the TRB CDR3 sequences (Huang et al., 2020). Therefore, analyzing TCR-seq data of Tscm and other memory subsets may provide novel perspectives for unveiling functions of Tscm.

We analyzed the repertoire features of the TRB repertoire in Tscm and Tm, including sequence composition (k-mer), gene segments, the TRB repertoire structure and CDR3 length distribution. We then unveil the differences of the antigen specificity between Tscm and Tm. We trained a SVM model with a large dataset (Emerson et al., 2017) to identify the public clonotypes in each sample, and showed that public clonotypes within top1000 abundant clonotypes in Tscm were more than those in Tm. The public clonotypes in Tscm have a different sequence composition comparing with public clonotypes in Tn. It confirms that the high abundant, public clonotypes in Tscm are antigen-experienced. We further used a sequence-based method to cluster clonotypes targeting same antigens, and showed that the public clonotypes in Tscm could recognize more antigens than those in other memory T cell subsets. Finally, we found more presence of similar clonotypes to those found in database and recognized autoreactive antigens in type one diabetes (T1D) patients.

## Materials and Methods

### Datasets

In this study, we conducted analyses on high-throughput TCR repertoire datasets of CD4^+^ T cell subsets. Gomez-Tourino et al. (2017) used a stringent strategy to sort Tn (CD3^+^CD4^+^CD45RO^−^CD27^+^CCR7^+^CD95^−^), Tscm (CD3^+^CD4^+^CD45RO^−^CD27^+^ CCR7^+^CD95^+^), and Tm (CD3^+^CD4^+^CD45RO^+^CD27^+^) from eight healthy subjects (HD) and eight T1D patients by fluorescence-activated cell sorting (FACS). Then RNA was extracted and sequenced in parallel. The sequence data is immuneACCESS format (https://clients.adaptivebiotech.com/pub/peakman-2017-naturecommunications). We examined the number of clonotypes in T1D and HD. As shown in Fig. S1, no significant difference was presented between T1D and HD in any subset.

To generating a model to identify public clonotypes which can occur in more than two individuals, we used datasets from Emerson et al. (2017) for training and testing a support vector machine (SVM) model. The dataset includes data of two cohorts, and can be found at https://clients.adaptivebiotech.com/pub/emerson-2017-natgen.

### Statistical analysis and plots

Statistical analyses were performed with R. The paired Willcox-ranked test was used to examine the difference between two groups. The Kruskal-Wallis rank-sum test was used to examine the differences among multiple groups, and then Nemenyi test was used for multiple comparisons. The *p* values of multiple tests were corrected by false discovery rate (FDR) method. A test with a *p* value < 0.05 was considered as a significance. The Spearman correlation method was used to examine the correlation between samples of two groups. Graphics were generated with R package ggplot2. Principal component analysis (PCA) was conducted with R package *forcats*. R package *readr*, *dplyr* and *tidyr* were used for statistics.

### Definition of a clonotype

A clonotype was defined as the amino acid sequence identity of the TRB CDR3 region.

### Determination of diversity

Renyi entropy was used in our study to evaluate the diversity with alpha value from 0 to 20. When alpha increases, clonotypes with a higher frequency will have a greater influence on the entropy. When alpha equal 0, the Renyi entropy is the logarithm of the number of clonotypes; When alpha is 1, Renyi entropy tends to the Shannon entropy. When the alpha approaches infinity, the Renyi entropy is determined by the most frequent clonotype, where a lower frequency of the most frequent clonotype will generate a higher Renyi entropy index.

Renyi entropy formula is

\begin{eqnarray*}H= \frac{1}{1-\alpha } \ln \nolimits \left( \sum _{i=1}^{n}{f}_{i}^{\alpha } \right) \end{eqnarray*}

Shannon diversity index formula is

\begin{eqnarray*}H=-\sum _{i=1}^{R}{p}_{i}\ln \nolimits {f}_{i} \end{eqnarray*}

where *H* is the diversity index, *α* is the alpha value, *n* is the total number of clonotypes, and *f*_*i*_ is the frequency of the *i*th clonotype.

### Hierarchical clustering

We performed ‘complete linkage’ clustering algorithm on the correlation matrix, and visualized dendrograms using *pheatmap*, a R package [50]. The Euclidean distance was used as a distance metric. Pearson correlation method was used to measure the correlation of TRB repertoire structures.

### SVM model for identifying public and private clonotypes

SVM analysis was performed using kernel-based analysis of biological sequences with the R package KeBABS (Palme, Hochreiter & Bodenhofer, 2015). Amino acid sequence of clonotypes was split into features with length *k* = 3. A cost parameter *C* = 100 was used for the misclassification of a sequence. A total of 320,000 public and 320,000 private clonotypes were randomly sampled from the total set, and then were split into training (80%) and test (20%) sets. SVM training was performed on the training set, and class prediction was performed on the test set. Prediction accuracy of classification was qualified by calculating $BACC= \frac{1}{2} \times \left( spec+sens \right) $, where specificity was calculated as $spec= \frac{TN}{TN+FP} $, and sensitivity was defined as $sens= \frac{TP}{TP+FN} $, (where TN = true negative, FP = false negative, TP = true positive and FN = false negative). The area under the receiver operating characteristic curve (AUC) was calculated, where the AUC = 0.5 means a random classification (BACC = 50%), and AUC = 1 means a perfect classification (BACC = 100%).

The dataset (Emerson et al., 2017) includes two cohorts: cohort 1 includes 666 individuals, and cohort 2 includes 116 individuals. We termed clonotypes occurred in no less than two individuals as public clones and ones occurred in only one individual as private clonotypes. Data of cohort 1 was used as a training set. For this dataset of cohort 1, we randomly sampled 20,000, 40,000, 80,000, 160,000 and 320,000 public clonotypes and an equal number of private clonotypes, train a SVM model, and tested the model by cross validation (Fig. S2). When sampling more than 160,000 public clonotypes, the increase in the sample size had limited improvement in model accuracy. To save computing source, we trained a model on the 320,000 public clonotypes and 320,000 private clonotypes. The model was then validated with data of cohort2. In order to further increase prediction accuracy, we tested two thresholds for public clonotype definition: (1) definition of public clonotypes occurred in at least three subjects; (2) definition of public clonotypes occurred in at least two subjects. Comparing to the second threshold, the definition of public clonotypes occurred in at least three subjects elevated the prediction accuracy from 82% to 88%. We therefore defined public clonotypes occurred in at least three subjects.

### Prediction of epitope specificity of clonotypes

GLIPH2 (Huang et al., 2020) is a robust tool to predict the cluster of clonotypes targeting the same epitope. Here, we used this method to cluster clonotypes recognizing the same antigens. The reference of CD4^+^ T clonotypes, the clonotypes gene usage and length distribution of CDR3 were included in ref_CD4.txt, ref_V_CD4.txt and ref_L_CD4.txt downloaded from the official website of GLIPH2 (http://50.255.35.37:8080/). A filter with a high stringency (Fisher_score < 0.0001, number of subjects >3, and number_unique_cdr3 ≥ 3) was used for identify the number of potential antigens in each sample.

## Results

### Tscm and Tm had different TRB repertoire structures

**Figure 1:**
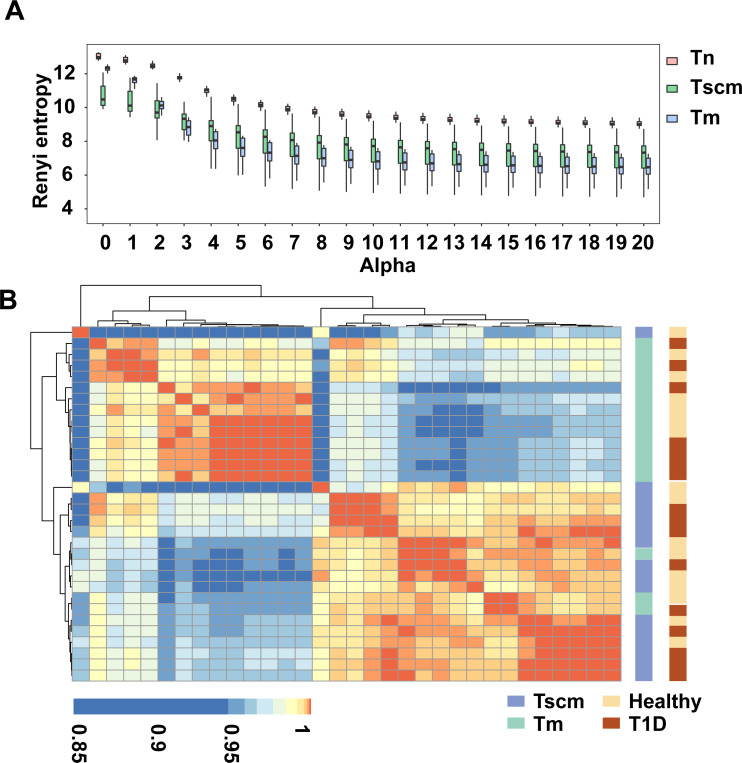
Tscm and Tm had different TRB repertoire structure. (A) The Renyi entropy of Tn, Tscm, and Tm with alpha values from 0 to 20 (step size of 1). The median, the first and third quartiles were shown. (B) The Renyi entropy profiles were hierarchically clustered based on Pearson correlation coefficient with an alpha range of 0 to 10 (step size of 0.2).

To characterize immune repertoire clonal structure, we used Renyi entropy (Greiff et al., 2015). The *α*-values represent weights, which means as *α* increases, higher frequency clonotypes are weighted more. For a given alpha value, a larger Renyi entropy means a more considerable diversity of the sample. Since each alpha value focuses on a different stretch of the immune repertoire, this method enabled the reliable capture of TRB repertoire clonal frequency distributions. Our results showed that as alpha value increased, the Renyi entropy of all memory subsets decreased. At all alpha values, the Renyi entropy of Tn was higher than that of both Tscm and Tm. At the alpha value from 0 to 2, the Renyi entropy of Tscm was less than that of Tm, while after alpha value 3, the Renyi entropy of Tscm was greater than that of Tm (Fig. 1A), which reflects that Tscm had a greater diversity than Tm in the abundant clonotypes. Since the Renyi entropy profiling can recover a large amount of immunodiagnostic fingerprints from TRB repertoire data, we used the Renyi entropy to classify the cell subsets with hierarchical clustering approach based on Pearson correlation (Greiff et al., 2015). The results showed that Tscm of 13 subjects were clustered together; Tm of 15 subjects were gathered; Tscm of only 3 subjects mixed in the cluster of Tm (Fig. 1B). This hierarchical clustering result suggested that Tscm and Tm had different TRB repertoire structures.

### Top1000 clonotypes of Tscm and Tm used different genes

**Figure 2:**
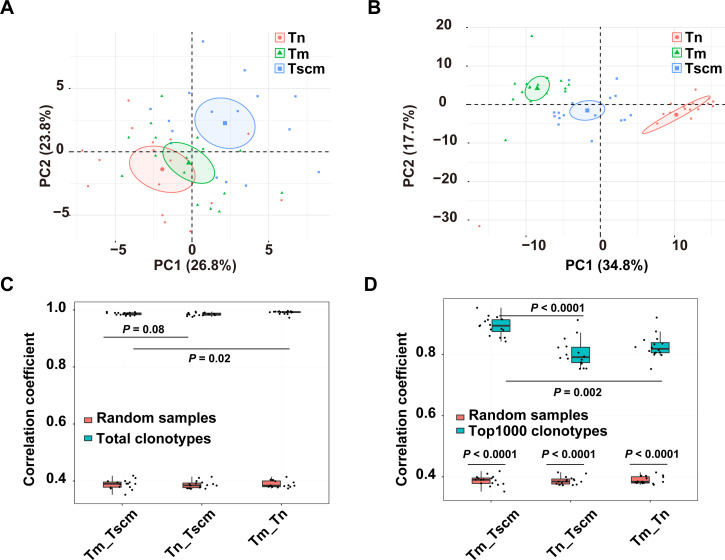
Tscm and Tm had distinct gene usage. (A) The PCA on the gene usage of the total clonotypes of Tn, Tscm, and Tm. Each dot represents one sample from a subject. (B) The PCA on the gene usage of the top1000 abundant clonotypes of Tn, Tscm, and Tm. Each dot represents one sample from a subject, each ellipse shows a 95% confidence ellipse, and the centroid presents the mean of PC1 as well as PC2 of samples in a cluster. (C) The Spearman correlation of the gene usage of the total clonotypes among Tn, Tscm, and Tm. The median, the first and third quartiles were shown. (D) The Spearman correlation of the gene usage of the top1000 abundant clonotypes among Tn, Tscm, and Tm. The median, the first and third quartiles were shown. For (A) to ( D), each dot represented a sample. The paired Wilcox-ranked test was used in (C) and (D), and the *p* values were corrected by FDR method.

The gene usage of the TRB repertoire of memory T cells is heavily affected by antigen experience. We analyzed the gene usage to unveil the effects of antigen-experience on Tscm and Tm, respectively. Since the frequent clonotypes of memory T cells are expanded by chronic antigen stimulations, we also analyzed the gene usage of the top1000 abundant clonotypes. The PCA on the gene usage of the entire repertoire separated Tn, Tscm, and Tm from each other (Fig. 2A). Specially, the PCA on the genes of the top1000 clonotypes could achieve a better performance of classification (Fig. 2B). A further analysis on top1000 abundant clonotypes showed that Tscm and Tm differentially used 12 V-genes and 3 J-genes: the frequency of TRBV12-03, TRBV07-09, TRBV18-01, TRBV23-01 and TRBJ02-07 were less in Tm than in Tscm; the frequency of TRBV03-01, TRBV02-01, TRBV11-02, TRBV09-01, TRBV06-05, TRBV25-01, TRBV24-01, TRBV05-05, TRBJ01-02 and TRBJ02-02 was greater in Tm than in Tscm (Table S1). In further, we used Spearman correlation to quantify the similarity of the gene usage between Tscm and Tm. Because Tscm and Tm differentiate from Tn, we therefore used the correlation between Tn and Tscm as a contrast. For the entire TRB repertoire, the correlation between Tm and Tscm was similar to that between Tscm and Tn, but less than that between Tm and Tn (Fig. 2C); for the top 1000 abundant clonotypes, the correlation between Tscm and Tm was greater than that between Tscm and Tn (Fig. 2D). It suggests that Tscm and Tm had a large difference in the gene usage of the TRB repertoire, especially in the range of top1000 abundant clonotypes.

### Tscm was different from Tm in CDR3 length distribution

**Figure 3:**
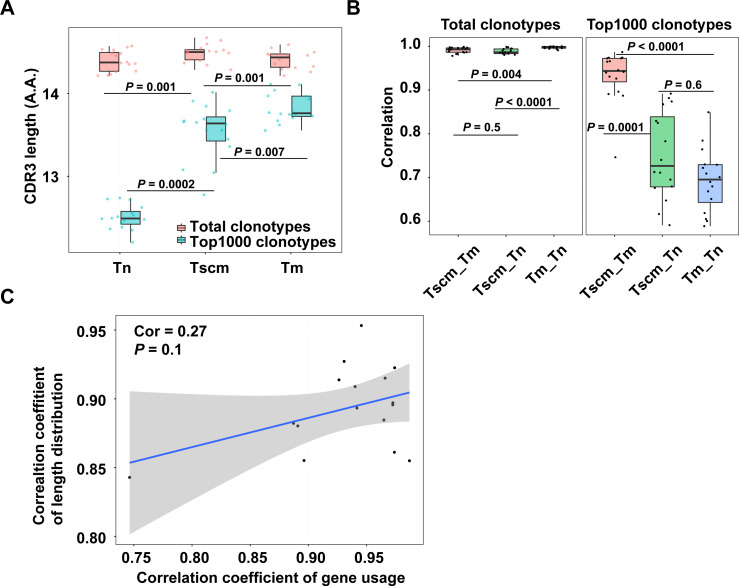
Tscm and Tm had different distributions of CDR3 length. (A) The mean CDR3 length of the total clonotypes (red), and the top1000 abundant clonotypes (blue) in Tn, Tscm, and Tm. The median, the first and third quartiles were shown. (B) The Spearman correlation coefficient of the distribution of CDR3 length of the total clonotypes (left), and top1000 abundant clonotypes (right) among Tn, Tm, and Tscm. The median, the first and third quartiles were shown. (C) the Spearman correlation calculated on the correlation coefficient of gene usage and the correlation coefficient of CDR3 length distribution in Tn, Tscm, and Tm. From A to C, each dot represents one sample from a subject. The Kruskal–Wallis rank-sum test was used to examine the difference among multiple groups in A and B, and then Nemenyi test was used for multiple comparisons.

The antigen experience has a selection on clonotypes which may change the distribution of CDR3 length. For the CDR3 of the total clonotypes, Tscm was significantly longer than Tn, and Tm as well (Fig. 3A). For the CDR3 of the top1000 abundant clonotypes, Tscm was obviously shorter than Tm, but longer than Tn. (Fig. 3A). According to the Spearman correlation analysis, Tscm had a similar length distribution to Tm rather than to Tn in the top1000 abundant clonotypes (Fig. 3B). Tscm and Tm also showed an increased correlation in gene usage, then we examined whether gene usage was related to the distribution of CDR3 length in Tscm and Tm. However, we did not find a high correlation (cor = 0.27) between the gene usage and distribution of CDR3 length in Tscm and Tm (Fig. 3C), suggesting that, between Tscm and Tm, the increased correlation of gene usage and elevated correlation of length distribution might be independent.

### Tscm had special CDR3 sequence compositions

**Figure 4:**
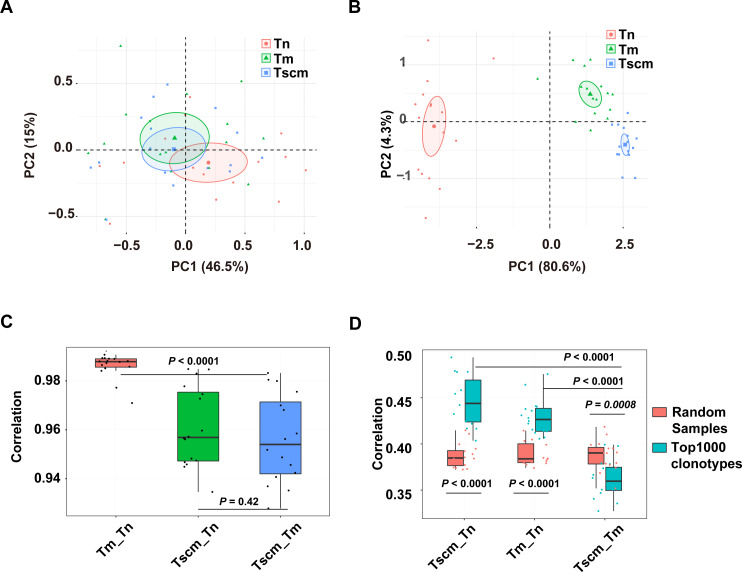
The sequence composition of the top1000 clonotypes of CD4^+^ T memory cell subsets and their correlations. (A) The principal component analysis (PCA) of the sequence composition (k-mer) of the entire clonotypes of Tn, Tscm, and Tm. Each dot represents one sample from a subject. (B) The PCA of the sequence composition (k-mer) of the top1000 abundant clonotypes of Tn, Tscm, and Tm. Each dot represents one sample from a subject, each ellipse shows a 95% confidence ellipse, and the centroid presents the mean of PC1 as well as PC2 of samples in a cluster. (C) The Spearman correlation coefficient of sequence composition of the entire clonotypes between Tn and Tscm, between Tm and Tn, and between Tm and Tscm. The median, the first, and third quartiles were shown. (D) The Spearman correlation coefficient of sequence composition of both top1000 abundant clonotypes (blue) and randomly sampled 1000 clonotypes (red) between Tn and Tscm, between Tm and Tn, and between Tm and Tscm. The median, the first, and third quartiles were shown. For C and D, the paired Wilcox-paired ranked test was used, and then the *p* values were corrected by the FDR method.

We examined the CDR3 sequence composition by decomposing kernels containing three amino acids. As showed by the PCA on the sequence composition of all clonotypes, Tn, Tm, and Tsm samples were partially separated (Fig. 4A). Exhibited by the PCA based on the k-mer of the top1000 abundant clonotypes, the samples of Tn, Tscm, and Tm were completely separated (Fig. 4B). These results suggested that Tscm and Tm had a great difference in the sequence composition of the top1000 clonotypes. We used Spearman correlation to quantify differences of sequence composition among subsets. For the total TRB repertoire, the correlation between Tm and Tscm was significantly weaker than that between Tn and Tm, and slightly weaker than that between Tscm and Tn (Fig. 4C). For the top1000 abundant clonotypes, the correlation between Tm and Tscm was significantly weaker than that between Tn and Tscm, and between Tn and Tm as well (Fig. 4D). Furthermore, to identify whether the correlation between Tscm and Tm reduced in the top1000 clonotypes, we randomly sampled 1,000 clonotypes from the entire repertoire as a contrast. Our results showed that the correlation coefficient of the top1000 clonotypes between Tscm and Tm was significantly lower than that of random subsamples. In contrast, the correlation of top1000 clonotypes sequence composition between Tn and Tscm, and that between Tn and Tm were significantly stronger than that of random samples between corresponding cell subsets (Fig. 4D). It suggested that Tscm and Tm were different in the sequence composition of the entire TRB repertoire, especially the top1000 abundant clonotypes.

### Frequent clonotypes of Tscm were more public

**Figure 5:**
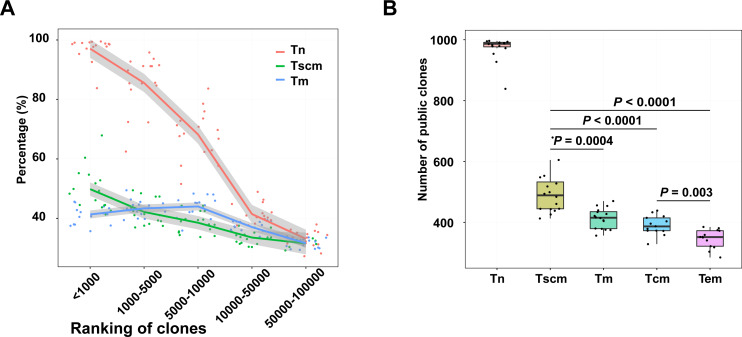
Top1000 clonotypes of Tscm were more public. (A) The percentage of public clonotypes within frequency ranges. (B) The percentage of public clonotypes within top1000 abundant clonotypes of Tn, Tscm, Tm, Tcm and Tem. The median, the first and third quartiles were shown. The Wilcox-ranked test was used in B, and the *p* values were corrected by FDR method. For A and B, each dot represented a sample.

Public clonotypes which are shared among subjects can be induced by gene recombination and antigen stimulation (Venturi et al., 2008). To evaluate the public clonotypes’ distribution in each sample, we trained a SVM model to identify public clonotypes with a large dataset (Greiff et al., 2017). This dataset includes two cohorts. The SVM model had a high prediction accuracy (BACC = 88% and AUC = 95%) in cohort 1 and presented a strong robustness when classifying public clonotypes on the data of cohort 2 (BACC = 82%) (Fig. S3). Using the SVM model, we identified public clonotypes from samples of Tn, Tscm, Tcm, and Tem. Since the public clonotypes may be unevenly distributed in different frequency ranges of the TRB repertoire, we calculated the percentage of public clonotypes for each frequency range. We ranked clonotypes by their frequency. Our result showed that as the ranks increased, the percentage of public clonotypes decreased, which was inconsistent with findings in global T cells (Soto et al., 2020). Notably, percentage of identified public top1000 clonotypes in Tscm were more than the percentage in Tm. (Fig. 5A). We also used the data of top1000 clonotypes of effector memory T cells and central memory T cells presented by Jiang et al. (2020), and found a similar trend that Tscm contained more public top1000 clonotypes than effector memory T cells (Tem) as well as central memory T cells (Tcm). (Fig. 5B). We further trained a SVM model to classify public clonotypes of Tn and Tm, and showed a prediction accuracy of 66%, which suggested that public clonotypes of Tn and public clonotypes of Tscm contained predictive high-dimensional features.

To further identify the antigen specificity of clonotypes in memory subsets, we used GLIPH2 to cluster clonotypes potentially targeting same antigens. A stringent cutoff was used to avoid potential mistakes when performed GLIPH2. In GLIPH2, a clonotype can be grouped in over one cluster. For top1000 abundant clonotypes, 1095 clusters were exhibited in Tn, 176 exhibited in Tscm, and 71 exhibited in Tm. For public clonotypes within top1000 clonotypes, we defined 541 clusters in Tn, 117 in Tscm, 67 in Tm (Tables S2 and S3).

### Self-reactivated clonotypes expanded in Tscm rather than Tm in T1D patients

**Figure 6:**
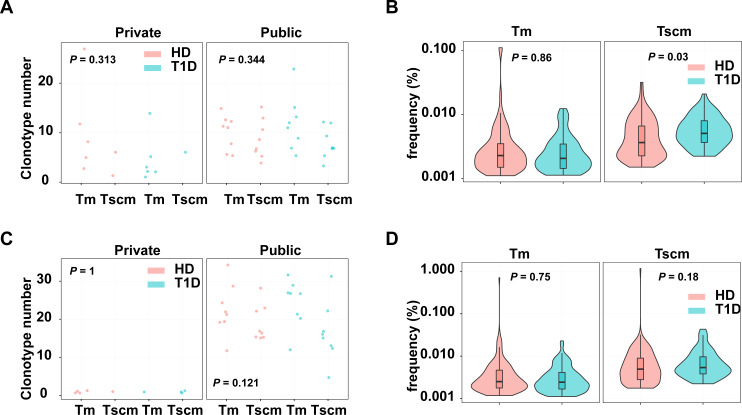
Tscm included more self-reactive clonotypes than Tm. (A) The number of GAD-related clonotypes in each subject of T1D and HD. (B) The frequency of GAD-related, public clonotypes in Tm, and Tscm of T1D and HD. (C) The number of pathogen-related clonotypes in each subject of T1D and HD. (D) The frequency of pathogen-related, public clonotypes in Tm, and Tscm of T1D and HD. The median, the first and third quartiles were shown in boxplot. The Kruskal–Wallis rank-sum test was used to examine the difference among multiple groups, and then Nemenyi test was used in multiple comparisons.

Since Tscm potentially play roles in infections and autoimmune diseases, we evaluated the pathogen and autoreactive clonotypes in Tscm and Tm, respectively. The clonotypes were annotated by a database including 1,885 pathogen-related clonotypes from VDJdb (Bagaev et al., 2020), 1,409 autoreactive clonotypes (Gomez-Tourino et al., 2017). Our results showed that autoreactive, private clonotypes could be found in Tscm of 11 subjects, and in Tm of 3 subjects; autoreactive, public clonotypes were shown in Tscm of 16 subjects, and in Tm of 16 subjects. The number of autoreactive clonotypes were similar in Tscm and Tm, however, there were more autoreactive public clonotypes than autoreactive private clonotypes in each sample (Fig. 6A). Considering clonotypes’ frequency in each subject, we showed that GAD-related clonotypes within Tscm became more frequent in T1D than in HD. In contrast, the frequency of GAD-related clonotypes in Tm was similar in T1D and HD (Fig. 6B). Meanwhile, we showed more pathogen-related public clonotypes than pathogen-related private clonotypes for Tscm and Tm (Fig. 6C). However, we did not find that pathogen-related clonotypes of either Tscm or Tm were more frequent in T1D (Fig. 6D). In conclusion, self-reactivated clonotypes expanded in Tscm of T1D patients.

## Discussion

Heterogeneous phenotypic and functions make it controversial to conclude that Tscm represent a stable subset (Cieri et al., 2015; Sallusto & Lanzavecchia, 2009). Studies in HSCT and on estimating Tscm turn-over rate indicated that Tscm were maintained by dynamic-influx from naïve. However, which clonotypes are selectively expanded during the differentiation from naïve is still unclear. Meanwhile, the dynamic of Tcm and Tem were also tracked in mice model, and about half of Tcm and Tem were long-lived and self-renewed, and the other part can be supplied by differentiation from naïve directly (Gossel et al., 2017). Thus, a question is raised whether the TCR repertoire of Tscm and other memory subsets are similar. In this study, we analyzed TRB repertoire data of Tscm from a previous study. Tscm presented different features of TRB clonotype compared with other memory subsets. These features included TRB repertoire structure, gene usage, CDR3 length, and sequence composition. In previous studies, top1000 clonotypes were different from those of naïve, which was considering as a result of antigen-driven selection. In this study, we found that top1000 clonotypes of Tscm have more overlap with naïve than other memory subsets. Furthermore, these differences between Tscm and Tm were greater than inter-individual differences within Tscm, implying that these TRB repertoire features could distinguish CD4^+^ Tscm and other CD4 ^+^ memory T subsets. Previously, Hou et al. (2020) reported that CD4+ memory T cells have a shortening distribution of CDR3 length and skewed gene usage compared with CD4^+^ naive cells. Hou et al. inferred that antigen experience leads the TRB repertoire of memory T cells skewed. Bilate et al. (2020) found that intestinal CD4^+^ intraepithelial lymphocytes differentiation was always accompanied by clonal restriction, suggesting that TCR and local antigen presentation is required by differentiation of this subset of T cells. Meyer-Olson et al. (2010) found that in HIV infections, TCR/peptide major histocompatibility complex interaction played a central role in the differentiation of TemRA cells (one type of effector memory T cells), but not in the differentiation of TemRO. Therefore, antigen experience affects T cell subsets’ differentiation differently, which lets TCR repertoire features divergent among T-cell subsets. This mechanism might explain the differences in TRB repertoire between Tscm and other CD4^+^ memory T cells. Besides, differentiation sequential is another factor influencing the TRB repertoire and is supported by some previous studies (Minervina et al., 2021; Minervina et al., 2020; Snook, Kim & Williams, 2018). Hogan et al. (2019) inferred that early exposure to self and environmental antigens establishes persistent memory populations at levels mainly determined by the dirtiness of the environment. After the first few weeks of life, new memory cells replace these populations at rates independent of the environment. Tcm showed a larger proportion replacement than Tem, which implied that Tcm was affected by naïve cells more than Tem. Our result exhibited that TRB repertoire features of Tscm, rather than Tm, are more like those of naïve, suggesting that subset with a lower differentiation would be affected by naïve subset more. This notion is consistent with the hierarchical model of human T cell differentiation (Gattinoni et al., 2011).

Renyi entropy is a classical method for estimating all stretches of a community. Greiff et al. introduced this method to grab features of TCR repertoire and verified that TCR repertoire structure features captured by this method could separate individuals with different healthy statuses (Greiff et al., 2015). In this study, our data suggested that Tscm were separated from Tm, whether individuals did or did not get T1D, suggesting that a large difference in TCR repertoire structure between Tscm and other memory subsets during cell differentiation. When alpha is 1, Renyi entropy tends to the Shannon entropy. Therefore, Shannon entropy, a index used in many studies, was contained in the estimation by Renyi entropy.

Public clonotypes are defined as ones that appeared in more than one individual. The traditional method for identifying public clonotypes needs a large cohort. Greiff et al. (2017) found that public clonotypes could be separated by the SVM method from private ones. Therefore, we trained an SVM model based on a large cohort and then used this model to identify public clonotypes in a small sample size. A limitation of the original study was that the model’s accuracy for identifying public TCR clonotypes was about 76% and needed to be improved. We found that a large cohort (Emerson et al., 2017) and increased the threshold (termed public clonotypes as ones occurred in more than two individuals) for identifying public clonotypes could increase the model’s accuracy (BBAC over 85%). Public clonotypes can be generated during gene recombination (Carey et al., 2017) and thymus selection (Khosravi-Maharlooei et al., 2019). Public clonotypes enriched in naïve are likely promiscuous in their peptide-binding (Carey et al., 2017), while ones in memory are more specific. Tscm had more public clonotypes than other memory T cell subsets shown in this study. This study showed that the sequence composition of public clonotypes is slightly different from those in naïve, with an SVM prediction accuracy of 66%. It suggested that public clonotypes in Tscm and Tn might have different sequence composition, and implied that public clonotypes in Tscm might undergo a selection driven by antigen stimulation. The results of the GLIPG2 analysis further verified this hypothesis. In this analysis, public clonotypes from top1000 clonotypes of Tn had 1095 clusters, while Tscm only had 176 clusters. It suggests that public clonotypes in Tscm had an increased specificity than those in Tn, which is a feature of antigen-experienced TRB repertoire.

Two Methods were involved in this study for estimating epitope specificity of clonotypes in subsets: GLIPH2 and estimation of enrichment of self-reactive clonotypes. GLIPH2 is based on a hypothesis that the sequence composition of TCR, especially the CDR3 region, determine the antigen specificity of clonotype. This model is trained on TCR clonotypes with known specificities. Huang et al. (2020) verified that GLIPH2 model could precisely cluster clonotypes recognizing the same epitope under a stringent threshold. Therefore, number of clusters predicted in our studies could reflect the number of potential epitopes recognized by clonotypes. As shown in our results, clusters of Tscm was less than naïve, but more than other memory subsets, suggesting that Tscm still maintained ability to recognize diverse antigens after antigen-specific stimulation. The other method was counting the frequency of self-reactive clonotypes in each individual. Because CDR3 primarily affected the antigen specificity of clonotype, accounting the frequency and percentage of self-reactive clonotypes identified in previous studies could reflect the functions of memory subsets. Infection and autoimmune related clonotypes in T1D and healthy groups were estimated respectively. The results confirmed the expansion of self-reactive clonotypes in Tscm rather than in other memory subsets in T1D patients.

The different TRB repertoire composition indicated a different function of Tscm compared with other memory T cell subsets. As shown by Greiff et al. (2017), public clones are usually generated by specific inserts and/or deletions (indel) during somatic hypermutation. Shown by other studies as well as our results, during cell differentiation, public clonotypes reduced in memory and effector. Although the cellular mechanism underlying this phenomenon is still unclear, the selection on TCR clonotypes during differentiation were wildly observed. Our study showed that top1000 clonotypes were more public in Tscm than in other memory subsets, suggesting that a characteristic of Tscm is enriching public clonotypes. Furthermore, self-reactive clonotypes were defined as ‘public’ in our study, suggesting that abnormal indels happened in these self-reactive clonotypes make them ’public’. Therefore, the ‘public’ feature contributed to the enrichment of self-reactive clonotypes in Tscm. Roberto et al. (2015) introduced that self/tumor-antigen specific TCRs were enriched in Tscm after HSCT, and Interluekin-7 was essential for Tscm generation (Cieri et al., 2013; Cieri et al., 2015). Thus, environment of Tscm generation might Tscm enrich ‘public’ clonotypes. The increased level of Tscm were also found in other studies focus on autoimmune diseases. For example, a study confirms that CD4^+^ Tscm recognizing citrullinated epitopes were expanded in rheumatoid arthritis (Cianciotti et al., 2020). The frequency of CD8^+^ Tscm was increased in acquired aplastic anemia (Hosokawa et al., 2016). Autoreactive CD8^+^ Tscm occurs in T1D (Vignali et al., 2018). Our results indicated the expansion of autoreactive clonotypes in T1D. These observations suggested that Tscm might serve as a pool for autoreactive clonotypes. Although the factors to lead the enrichment of self-reactive clonotypes in Tscm is still unclear, our data showed that it might be possible to amiable T1D by targeting Tscm in T1D patients. As shown by Ahmed et al. (2016), Tscm highly expressed Ki67, indicating an activated status of Tscm. Therefore, therapies suppressing immune activation may be possible to attenuate the pathology of T1D.

##  Supplemental Information

10.7717/peerj.11987/supp-1 Supplemental Information 1 Statistics of clonotypes numbers for each T-cell susbet of T1Ds and HDs Each data point indicates an individual. The median line indicates the mean value, the upper line of the error bar inidicates 75% of values, and lower line of error indicates 25% of values. The Wilcox-ranked test was used to examine significance of intra-groups differneces, and then *p* values were corrected by false discovery rate approach.

10.7717/peerj.11987/supp-2 Supplemental Information 2 Training a SVM model for classifying public clones (A) The prediction accuracy (BACC) of the model training based on 20000, 40000, 80000, 160000, 320000 public clones and an equal number of private clones. The public clones were defined as clones occurred in at least three subjects. The sequences were split into training (80%) and test (20%) sets. (B) The BACC of the model training based on 320000 public clones and an equal number of private clones. For red, the public clones were defined as clones occurred in at least three subjects. For blue, the public clones were defined as clones occurred in at least two subjects. The Wilcox-ranked test was used in B.

10.7717/peerj.11987/supp-3 Supplemental Information 3 The PCA on the gene usage of the top1000 abundant clones of Tn, Tcm, and Tem Each dot represents one sample from a subject, each ellipse shows a 95% confidence ellipse, and the centroid presents the mean of PC1 as well as PC2 of samples in a cluster.

10.7717/peerj.11987/supp-4 Supplemental Information 4 The gene usage of top1000 abundant clonotypes of Tscm and Tm

10.7717/peerj.11987/supp-5 Supplemental Information 5 The GLIPH2 results of Tscm top1000 clonotypes

10.7717/peerj.11987/supp-6 Supplemental Information 6 The GLIPH2 results of Tm top1000 clonotypes

## Additional Information and Declarations

### Data Availability

The following information was supplied regarding data availability: Data is available at immuneACCESS: B7C88S, B7001Z. https://clients.adaptivebiotech.com/pub/peakman-2017-naturecommunications. https://clients.adaptivebiotech.com/pub/emerson-2017-natgen.

## References

[ref-1] Ahmed R, Roger L, Costa Del Amo P, Miners KL, Jones RE, Boelen L, Fali T, Elemans M, Zhang Y, Appay V, Baird DM, Asquith B, Price DA, Macallan DC, Ladell K (2016). Human stem cell-like memory T cells are maintained in a state of dynamic flux. Cell Reports.

[ref-2] Bagaev DV, Vroomans RMA, Samir J, Stervbo U, Rius C, Dolton G, Greenshields-Watson A, Attaf M, Egorov ES, Zvyagin IV, Babel N, Cole DK, Godkin AJ, Sewell AK, Kesmir C, Chudakov DM, Luciani F, Shugay M (2020). VDJdb in 2019: database extension, new analysis infrastructure and a T-cell receptor motif compendium. Nucleic Acids Research.

[ref-3] Barsoumian HB, Batra L, Shrestha P, Bowen WS, Zhao H, Egilmez NK, Gomez-Gutierrez JG, Yolcu ES, Shirwan H (2019). A novel form of 4-1BBL prevents cancer development via nonspecific activation of CD4 T and natural killer cells. Cancer Research.

[ref-4] Batra L, Shrestha P, Zhao H, Woodward KB, Togay A, Tan M, Grimany-Nuno O, Malik MT, Coronel MM, Garcia AJ, Shirwan H, Yolcu ES (2020). Localized immunomodulation with PD-L1 results in sustained survival and function of allogeneic islets without chronic immunosuppression. Journal of Immunology.

[ref-5] Bilate AM, London M, Castro TBR, Mesin L, Bortolatto J, Kongthong S, Harnagel A, Victora GD, Mucida D (2020). T cell receptor is required for differentiation, but not maintenance, of intestinal CD4(+) intraepithelial lymphocytes. Immunity.

[ref-6] Cao J, Zhang C, Han X, Cheng H, Chen W, Qi K, Qiao J, Sun Z, Wu Q, Zeng L, Niu M, Li L, Xu K (2019). Emerging role of stem cell memory-like T cell in immune thrombocytopenia. ScandInavian Journal of Immunology.

[ref-7] Carey AJ, Hope JL, Mueller YM, Fike AJ, Kumova OK, Van Zessen DBH, Steegers EAP, Burg Mvander, Katsikis PD (2017). Public clonotypes and convergent recombination characterize the naïve CD8+ T-cell receptor repertoire of extremely preterm neonates. Frontiers in Immunology.

[ref-8] Cianciotti BC, Ruggiero E, Campochiaro C, Oliveira G, Magnani ZI, Baldini M, Doglio M, Tassara M, Manfredi AA, Baldissera E, Ciceri F, Cieri N, Bonini C (2020). CD4+ memory stem T cells recognizing citrullinated epitopes are expanded in patients with rheumatoid arthritis and sensitive to tumor necrosis factor blockade. Arthritis & Rheumatology.

[ref-9] Cieri N, Camisa B, Cocchiarella F, Forcato M, Oliveira G, Provasi E, Bondanza A, Bordignon C, Peccatori J, Ciceri F, Lupo-Stanghellini MT, Mavilio F, Mondino A, Bicciato S, Recchia A, Bonini C (2013). IL-7 and IL-15 instruct the generation of human memory stem T cells from naive precursors. Blood.

[ref-10] Cieri N, Oliveira G, Greco R, Forcato M, Taccioli C, Cianciotti B, Valtolina V, Noviello M, Vago L, Bondanza A, Lunghi F, Marktel S, Bellio L, Bordignon C, Bicciato S, Peccatori J, Ciceri F, Bonini C (2015). Generation of human memory stem T cells after haploidentical T-replete hematopoietic stem cell transplantation. Blood.

[ref-11] Devarajan P, Chen Z (2013). Autoimmune effector memory T cells: the bad and the good. Immunologic Research.

[ref-12] Emerson RO, De Witt WS, Vignali M, Gravley J, Hu JK, Osborne EJ, Desmarais C, Klinger M, Carlson CS, Hansen JA, Rieder M, Robins HS (2017). Immunosequencing identifies signatures of cytomegalovirus exposure history and HLA-mediated effects on the T cell repertoire. Nature Genetics.

[ref-13] Fearon DT, Carr JM, Telaranta A, Carrasco MJ, Thaventhiran JED (2006). The rationale for the IL-2-independent generation of the self-renewing central memory CD8+ T cells. Immunological Reviews.

[ref-14] Flynn JK, Gorry PR (2014). Stem memory T cells (TSCM)-their role in cancer and HIV immunotherapies. Clinical & Translational Immunology.

[ref-15] Gao K, Chen L, Zhang Y, Zhao Y, Wan Z, Wu J, Lin L, Kuang Y, Lu J, Zhang X, Tian L, Liu X, Qiu X (2019). Germline-encoded tcr-mhc contacts promote TCR V gene bias in umbilical cord blood T cell repertoire. Frontiers in Immunology.

[ref-16] Gattinoni L, Lugli E, Ji Y, Pos Z, Paulos CM, Quigley MF, Almeida JR, Gostick E, Yu Z, Carpenito C, Wang E, Douek DC, Price DA, June CH, Marincola FM, Roederer M, Restifo NP (2011). A human memory T cell subset with stem cell-like properties. Nature Medicine.

[ref-17] Gattinoni L, Speiser DE, Lichterfeld M, Bonini C (2017). T memory stem cells in health and disease. Nature Medicine.

[ref-18] Gomez-Tourino I, Kamra Y, Baptista R, Lorenc A, Peakman M (2017). T cell receptor beta-chains display abnormal shortening and repertoire sharing in type 1 diabetes. Nature Communications.

[ref-19] Gossel G, Hogan T, Cownden D, Seddon B, Yates AJ (2017). Memory CD4 T cell subsets are kinetically heterogeneous and replenished from naive T cells at high levels. Elife.

[ref-20] Greiff V, Bhat P, Cook SC, Menzel U, Kang W, Reddy ST (2015). A bioinformatic framework for immune repertoire diversity profiling enables detection of immunological status. Genome Medicine.

[ref-21] Greiff V, Weber CR, Palme J, Bodenhofer U, Miho E, Menzel U, Reddy ST (2017). Learning the high-dimensional immunogenomic features that predict public and private antibody repertoires. Journal of Immunology.

[ref-22] Hogan T, Nowicka M, Cownden D, Pearson CF, Yates AJ, Seddon B (2019). Differential impact of self and environmental antigens on the ontogeny and maintenance of CD4(+) T cell memory. Elife.

[ref-23] Hosokawa K, Muranski P, Feng X, Townsley DM, Liu B, Knickelbein J, Keyvanfar K, Dumitriu B, Ito S, Kajigaya S, Taylor JG, Kaplan MJ, Nussenblatt RB, Barrett AJ, O’Shea J, Young NS (2016). Memory stem T cells in autoimmune disease: high frequency of circulating CD8+ memory stem cells in acquired aplastic anemia. The Journal of Immunology.

[ref-24] Hou X, Chen W, Zhang X, Wang G, Chen J, Zeng P, Fu X, Zhang Q, Liu X, Diao H (2020). Preselection TCR repertoire predicts CD4 and CD8 T-cell differentiation state. Immunology.

[ref-25] Huang H, Wang C, Rubelt F, Scriba TJ, Davis MM (2020). Analyzing the Mycobacterium tuberculosis immune response by T-cell receptor clustering with GLIPH2 and genome-wide antigen screening. Nature Biotechnology.

[ref-26] Jiang X, Wang S, Zhou C, Wu J, Jiao Y, Lin L, Lu X, Yang B, Zhang W, Xiao X, Li Y, Wu X, Wang X, Chen H, Zhao L, Fei Y, Yang H, Zhang W, Zhang F, Chen H, Zhang J, Li B, Yang H, Wang J, Liu X, Zhang X (2020). Comprehensive TCR repertoire analysis of CD4(+) T-cell subsets in rheumatoid arthritis. Journal of Autoimmunity.

[ref-27] Jimbo K, Konuma T, Watanabe E, Kohara C, Mizukami M, Nagai E, Oiwa-Monna M, Mizusawa M, Isobe M, Kato S, Takahashi S, Tojo A (2019). T memory stem cells after allogeneic haematopoietic cell transplantation: unique long-term kinetics and influence of chronic graft-versus-host disease. British Journal of Haematology.

[ref-28] Khosravi-Maharlooei M, Obradovic A, Misra A, Motwani K, Holzl M, Seay HR, De Wolf S, Nauman G, Danzl N, Li H, Ho S-H, Winchester R, Shen Y, Brusko TM, Sykes M (2019). Crossreactive public TCR sequences undergo positive selection in the human thymic repertoire. The Journal of Clinical Investigation.

[ref-29] Koch H, Starenki D, Cooper SJ, Myers RM, Li Q (2018). powerTCR: a model-based approach to comparative analysis of the clone size distribution of the T cell receptor repertoire. PLOS Computational Biology.

[ref-30] Krishna C, Chowell D, Gonen M, Elhanati Y, Chan TA (2020). Genetic and environmental determinants of human TCR repertoire diversity. Immunity & Ageing.

[ref-31] Lee YJ, Park JA, Kwon H, Choi YS, Jung KC, Park SH, Lee EB (2018). Role of stem cell-like memory T cells in systemic lupus erythematosus. Arthritis & Rheumatology.

[ref-32] Liu X, Zhang W, Zhao M, Fu L, Liu L, Wu J, Luo S, Wang L, Wang Z, Lin L, Liu Y, Wang S, Yang Y, Luo L, Jiang J, Wang X, Tan Y, Li T, Zhu B, Zhao Y, Gao X, Wan Z, Huang C, Fang M, Li Q, Peng H, Liao X, Chen J, Li F, Ling G, Zhao H, Luo H, Xiang Z, Liao J, Liu Y, Yin H, Long H, Wu H, Yang H, Wang J, Lu Q (2019). T cell receptor β repertoires as novel diagnostic markers for systemic lupus erythematosus and rheumatoid arthritis. Annals of the Rheumatic Diseases.

[ref-33] Lugli E, Dominguez MH, Gattinoni L, Chattopadhyay PK, Bolton DL, Song K, Klatt NR, Brenchley JM, Vaccari M, Gostick E, Price DA, Waldmann TA, Restifo NP, Franchini G, Roederer M (2013). Superior T memory stem cell persistence supports long-lived T cell memory. The Journal of Clinical Investigation.

[ref-34] MacLeod MK, Clambey ET, Kappler JW, Marrack P (2009). CD4 memory T cells: what are they and what can they do?. Seminars in Immunology.

[ref-35] Meyer-Olson D, Simons BC, Conrad JA, Smith RM, Barnett L, Lorey SL, Duncan CB, Ramalingam R, Kalams SA (2010). Clonal expansion and TCR-independent differentiation shape the HIV-specific CD8+ effector-memory T-cell repertoire in vivo. Blood.

[ref-36] Minervina AA, Komech EA, Titov A, Koraichi MBensouda, Rosati E, Mamedov IZ, Franke A, Efimov GA, Chudakov DM, Mora T, Walczak AM, Lebedev YB, Pogorelyy MV (2021). Longitudinal high-throughput TCR repertoire profiling reveals the dynamics of T-cell memory formation after mild COVID-19 infection. Elife.

[ref-37] Minervina AA, Pogorelyy MV, Komech EA, Karnaukhov VK, Bacher P, Rosati E, Franke A, Chudakov DM, Mamedov IZ, Lebedev YB, Mora T, Walczak AM (2020). Primary and secondary anti-viral response captured by the dynamics and phenotype of individual T cell clones. Elife.

[ref-38] Oakes T, Heather JM, Best K, Byng-Maddick R, Husovsky C, Ismail M, Joshi K, Maxwell G, Noursadeghi M, Riddell N, Ruehl T, Turner CT, Uddin I, Chain B (2017). Quantitative characterization of the T cell receptor repertoire of naive and memory subsets using an integrated experimental and computational pipeline which is robust, economical, and versatile. Frontiers in Immunology.

[ref-39] Palme J, Hochreiter S, Bodenhofer U (2015). KeBABS: an R package for kernel-based analysis of biological sequences. Bioinformatics.

[ref-40] Posnett DN (1995). Environmental and genetic factors shape the human T-cell receptor repertoire. Annals of the New York Academy of Sciences.

[ref-41] Qi Q, Liu Y, Cheng Y, Glanville J, Zhang D, Lee J-Y, Olshen RA, Weyand CM, Boyd SD, Goronzy JJ (2014). Diversity and clonal selection in the human T-cell repertoire. Proceedings of the National Academy of Sciences of the United States of America.

[ref-42] Roberto A, Castagna L, Zanon V, Bramanti S, Crocchiolo R, McLaren JE, Gandolfi S, Tentorio P, Sarina B, Timofeeva I, Santoro A, Carlo-Stella C, Bruno B, Carniti C, Corradini P, Gostick E, Ladell K, Price DA, Roederer M, Mavilio D, Lugli E (2015). Role of naive-derived T memory stem cells in T-cell reconstitution following allogeneic transplantation. Blood.

[ref-43] Sallusto F, Lanzavecchia A (2009). Heterogeneity of CD4+ memory T cells: functional modules for tailored immunity. European Journal of Immunology.

[ref-44] Seita J, Weissman IL (2010). Hematopoietic stem cell: self-renewal versus differentiation. Wiley Interdisciplinary Reviews: Systems Biology and Medicine.

[ref-45] Simons BD, Clevers H (2011). Strategies for homeostatic stem cell self-renewal in adult tissues. Cell.

[ref-46] Snook JP, Kim C, Williams MA (2018). TCR signal strength controls the differentiation of CD4 effector and memory T cells. Science Immunology.

[ref-47] Soto C, Bombardi RG, Kozhevnikov M, Sinkovits RS, Chen EC, Branchizio A, Kose N, Day SB, Pilkinton M, Gujral M, Mallal S, Crowe Jr JE (2020). High frequency of shared clonotypes in human T cell receptor repertoires. Cell Reports.

[ref-48] Stemberger C, Neuenhahn M, Gebhardt FE, Schiemann M, Buchholz VR, Busch DH (2009). Stem cell-like plasticity of naïve and distinct memory CD8+ T cell subsets. Seminars in Immunology.

[ref-49] Venturi V, Price DA, Douek DC, Davenport MP (2008). The molecular basis for public T-cell responses?. Nature Reviews Immunology.

[ref-50] Vignali D, Cantarelli E, Bordignon C, Canu A, Citro A, Annoni A, Piemonti L, Monti P (2018). Detection and characterization of CD8 autoreactive memory stem T Cells in patients with type 1 diabetes. Diabetes.

[ref-51] Zhang H, Liu L, Zhang J, Chen J, Ye J, Shukla S, Qiao J, Zhan X, Chen H, Wu CJ, Fu Y-X, Li B (2020). Investigation of antigen-specific T-cell receptor clusters in human cancers. Clinical Cancer Research.

