# Supplementary figures and images for: Characteristics of T-cell receptor repertoire of stem cell-like memory CD4+ T cells
Source: PeerJ. 2021 Aug 25;9:e11987. doi: 10.7717/peerj.11987 (PMC8401816; doi:10.7717/peerj.11987)

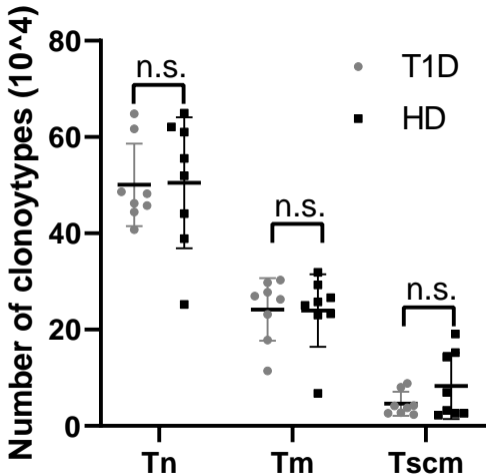

Supplement: Supplemental Information 1 — Each data point indicates an individual. The median line indicates the mean value, the upper line of the error bar inidicates 75% of values, and lower line of error indicates 25% of values. The Wilcox-ranked test was used to examine significance of intra-groups differneces, and then p values were corrected by false discovery rate approach. [file peerj-09-11987-s001.pdf]

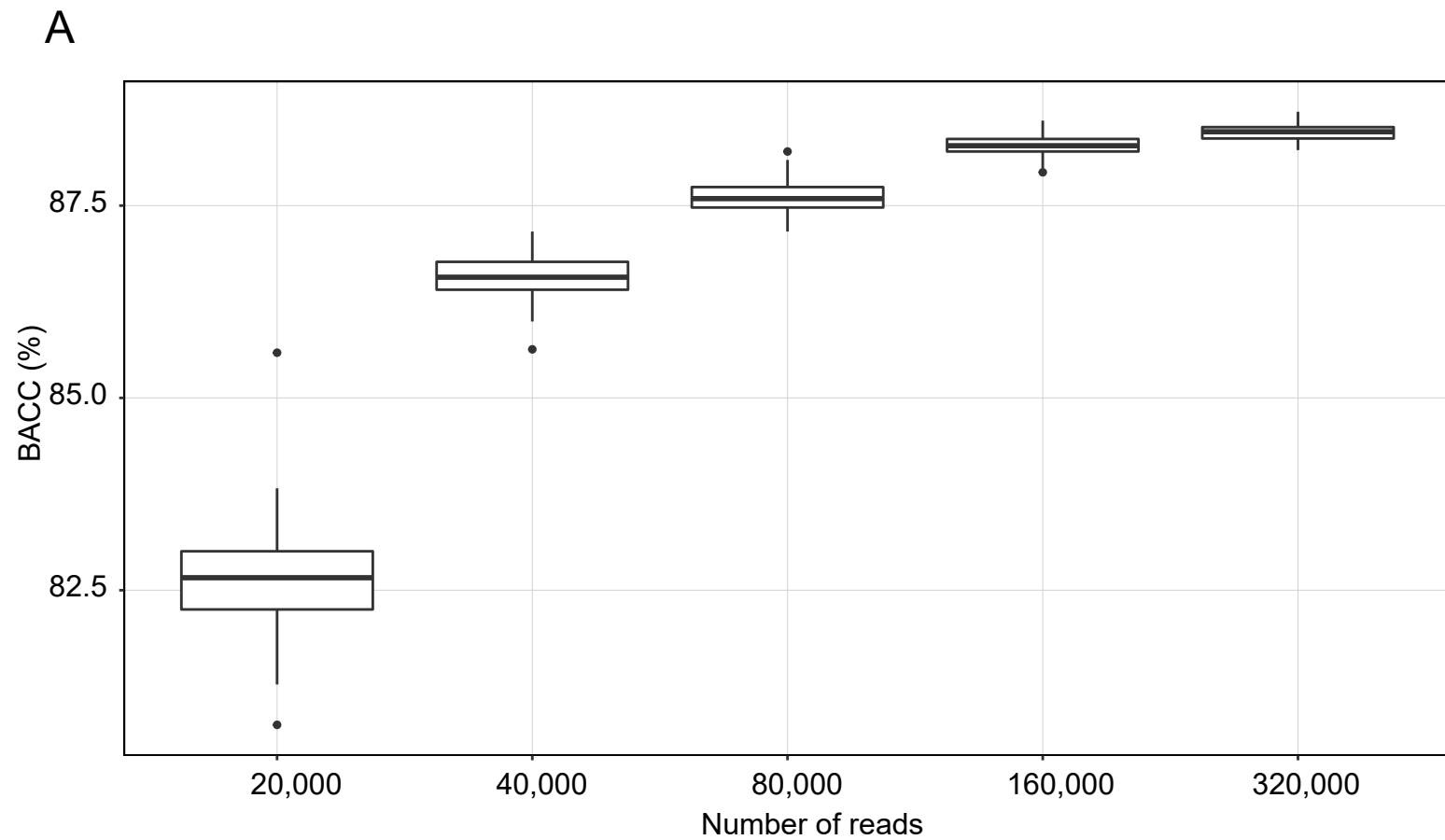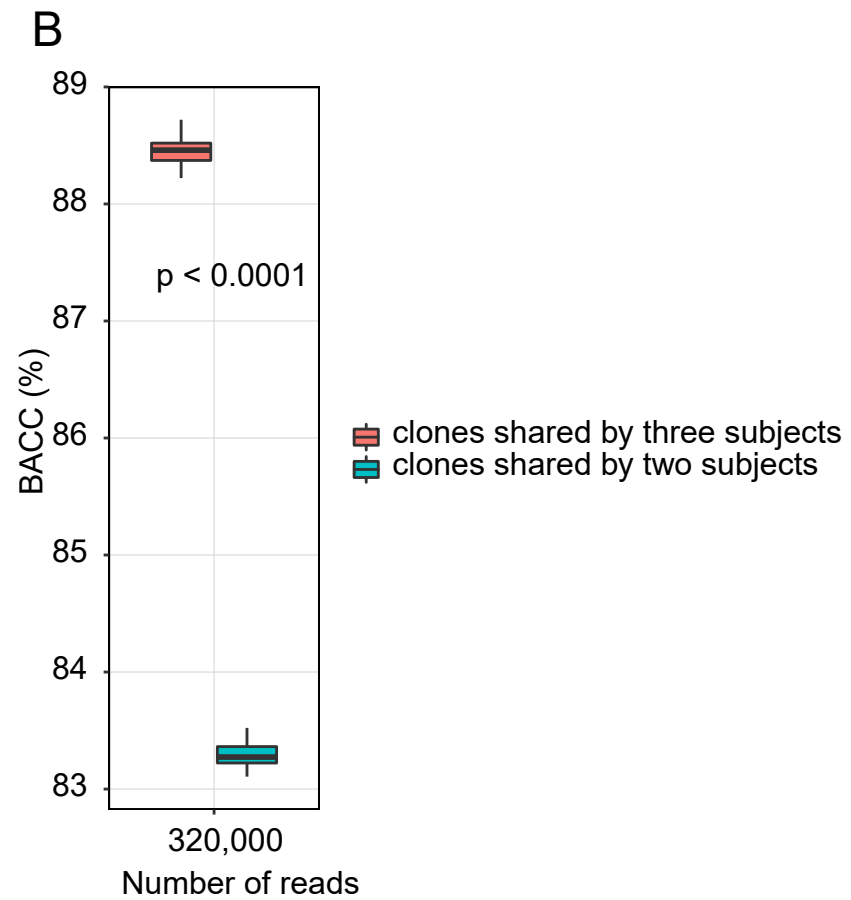

Supplement: Supplemental Information 2 — (A) The prediction accuracy (BACC) of the model training based on 20000, 40000, 80000, 160000, 320000 public clones and an equal number of private clones. The public clones were defined as clones occurred in at least three subjects. The sequences were split into training (80%) and test (20%) sets. (B) The BACC of the model training based on 320000 public clones and an equal number of private clones. For red, the public clones were defined as clones occurred in at least three subjects. For blue, the public clones were defined as clones occurred in at least two subjects. The Wilcox-ranked test was used in B. [file peerj-09-11987-s002.pdf]

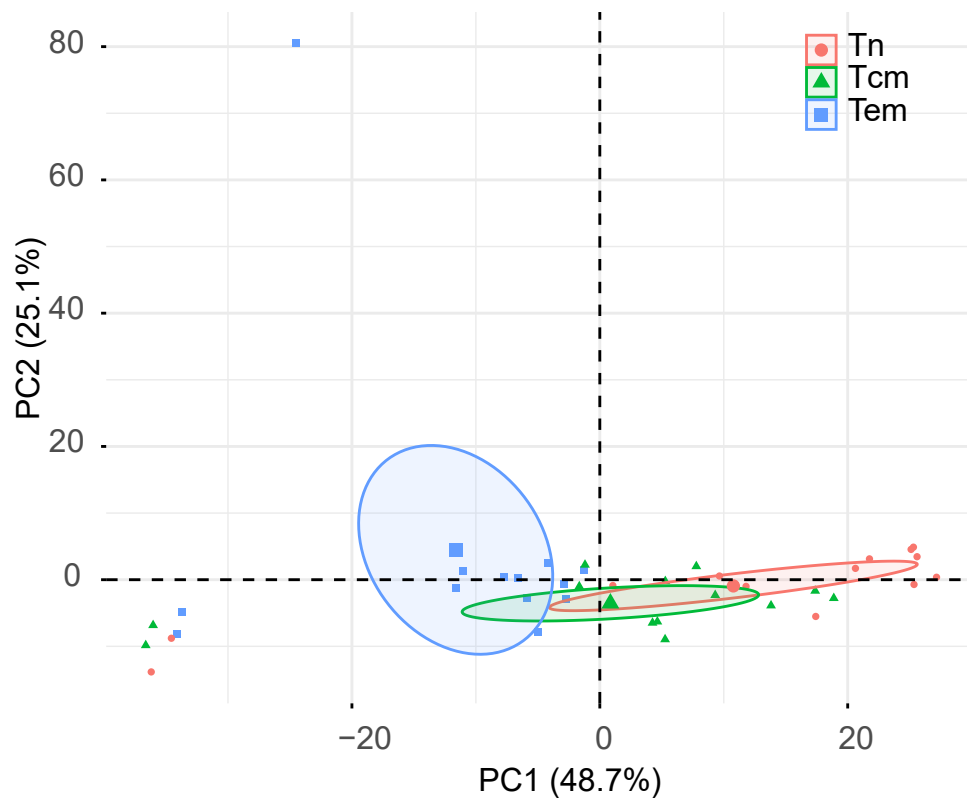

Supplement: Supplemental Information 3 — Each dot represents one sample from a subject, each ellipse shows a 95% confidence ellipse, and the centroid presents the mean of PC1 as well as PC2 of samples in a cluster. [file peerj-09-11987-s003.pdf]
